# *Tetranychus urticae* mites do not mount an induced immune response against bacteria

**DOI:** 10.1098/rspb.2017.0401

**Published:** 2017-06-07

**Authors:** Gonçalo Santos-Matos, Nicky Wybouw, Nelson E. Martins, Flore Zélé, Maria Riga, Alexandre B. Leitão, John Vontas, Miodrag Grbić, Thomas Van Leeuwen, Sara Magalhães, Élio Sucena

**Affiliations:** 1 Instituto Gulbenkian de Ciência, Rua da Quinta Grande, 6 2780-156 Oeiras, Portugal; 2 cE3c: Centre for Ecology, Evolution and Environmental Changes, Faculdade de Ciências, Universidade de Lisboa, Campo Grande, 1749-016 Lisbon, Portugal; 3 Laboratory for Agrozoology, Department of Crop Protection, University of Ghent, Coupure links 653, 9000 Ghent, Belgium; 4 Institute for Biodiversity and Ecosystem Dynamics, University of Amsterdam, Science Park 904, 1098 XH Amsterdam, The Netherlands; 5 Faculty of Applied Biotechnology and Biology, Department of Biology, University of Crete, Vasilika Vouton, PO Box 2208, 71409 Heraklion, Crete, Greece; 6 Institute of Molecular Biology and Biotechnology, Foundation for Research and Technology Hellas, 100 N. Plastira Street, 70013 Heraklion, Crete, Greece; 7 Laboratory of Pesticide Science, Department of Crop Science, Agricultural University of Athens, 75 Iera Odos Street, 11855 Athens, Greece; 8 Department of Biology, University of Western Ontario, London, Canada N6A 5B7; 9 Instituto de Ciencias de la Vid y del Vino Consejo Superior de Investigaciones Cientificas, Universidad de la Rioja, 26006 Logroño, Spain; 10 Departamento de Biologia Animal, Faculdade de Ciências, Universidade de Lisboa, Campo Grande, 1749-016 Lisboa, Portugal

**Keywords:** host–parasite interactions, *Tetranychus urticae*, *Sancassania berlesei*, microbiota, immunity

## Abstract

The genome of the spider mite *Tetranychus urticae*, a herbivore, is missing important elements of the canonical *Drosophila* immune pathways necessary to fight bacterial infections. However, it is not known whether spider mites can mount an immune response and survive bacterial infection. In other chelicerates, bacterial infection elicits a response mediated by immune effectors leading to the survival of infected organisms. In *T. urticae*, infection by either *Escherichia coli* or *Bacillus megaterium* did not elicit a response as assessed through genome-wide transcriptomic analysis. In line with this, spider mites died within days even upon injection with low doses of bacteria that are non-pathogenic to *Drosophila*. Moreover, bacterial populations grew exponentially inside the infected spider mites. By contrast, *Sancassania berlesei*, a litter-dwelling mite, controlled bacterial proliferation and resisted infections with both Gram-negative and Gram-positive bacteria lethal to *T. urticae*. This differential mortality between mite species was absent when mites were infected with heat-killed bacteria. Also, we found that spider mites harbour in their gut 1000-fold less bacteria than *S. berlesei*. We show that *T. urticae* has lost the capacity to mount an induced immune response against bacteria, in contrast to other mites and chelicerates but similarly to the phloem feeding aphid *Acyrthosiphon pisum*. Hence, our results reinforce the putative evolutionary link between ecological conditions regarding exposure to bacteria and the architecture of the immune response.

## 1. Introduction

To deal with infection, arthropods rely on several defensive mechanisms that include behavioural avoidance, physical and chemical barriers, and the immune response [1,2]. For example, virtually all arthropods studied thus far mount some combination of cellular and humoural responses against bacteria that rely on coagulation, production of reactive oxygen species (ROS), melanization, phagocytosis and the synthesis of antimicrobial peptides (AMPs) and/or enzymes [3,4].

In the insect model system *Drosophila*, the humoural response has been dissected genetically in great detail. It relies strongly on the induction of two signalling pathways, Toll and Imd, through the recognition of Lys-type or diaminopimelic acid (DAP) type peptidoglycans, present in Gram-positive and Gram-negative bacteria, respectively, and culminating in the production of AMPs [3,5].

Genomic analyses of other holometabolous insects have revealed that most genes of the Toll and Imd pathways are conserved, namely in mosquitoes, the honeybee and the beetle *Tribolium castaneum* [6–8]. However, in the pea aphid *Acyrthosiphon pisum*, a hemimetabolous insect, the Imd pathway is incomplete and several genes coding for receptors and common AMPs could not be identified. Moreover, this aphid species does not mount an immune response to bacterial infection [9,10]. Yet, this is not a general feature of hemipterans, because the Toll and Imd pathways along with several receptors and AMPs were annotated in the genome of the brown planthopper, *Nilaparvata lugens*, and several Toll pathway genes were shown to be upregulated upon bacterial infection in this species [11]. This pattern is also verified in another closely related hemipteran, *Rhodnius prolixus*, in which activity of the Imd pathway was experimentally confirmed [12]. Furthermore, *Imd* has been found in the genomes of other hemipterans such as the large milkweed bug (*Oncopeltus fasciatus*) and of the water strider *Gerris buenoi* (M van der Zee 2015, personal communication). Taken together, these observations suggest that, to a great extent, the immune response in most insects is directly comparable to that of the dipteran *Drosophila*.

In chelicerates, however, the Imd pathway seems to be incomplete in all species thus far analysed [13,14]. Notwithstanding, in *Carcinoscorpius rotundicauda*, an orthologue of the *Drosophila* NF-κB-like transcription factors, Relish, has been described and implicated in the immune response against *Pseudomonas aeruginosa* infection [15,16]. In fact, in several studied chelicerates, a response is elicited through the canonical production of antimicrobial compounds [17,18].

The two-spotted spider mite *Tetranychus urticae* feeds on the cell contents of a multitude of plant species. Its genome annotation failed to identify several canonical immunity genes, amongst which an important part of the Imd pathway and effectors such as haemolectins (von Willebrand factor-like proteins) or defensins [13,14,19] (electronic supplementary material, table S1). Two general hypotheses may explain this observation: (i) the spider mite mounts an immune response based on a different genetic basis, as do other chelicerates or (ii) as in aphids, *T. urticae* does not possess an inducible anti-bacterial immune response.

To distinguish between these hypotheses, we present experimental data describing the response of mites to bacterial systemic infection, including host survival, bacterial proliferation in the host and transcriptional responses. Additionally, we tested the generality of our results by repeating this characterization on the litter-dwelling grain mite *Sancassania berlesei* [20].

## 2. Results

### (a) *Tetranychus urticae* is susceptible to infection with *Escherichia coli* and *Bacillus megaterium*

**Figure 1.**
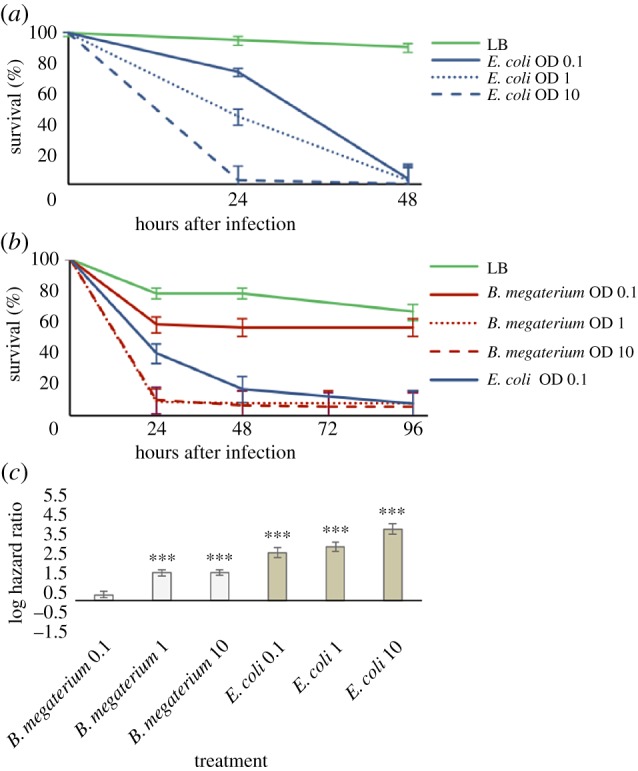
Bacterial infection severely affects survival of *T. urticae*. (*a*) *T. urticae* adult females were infected with *E. coli* at ODs 0.1, 1 and 10 and with LB as control. There is a clear reduction in survival of *T. urticae* after infection, independently of the bacteria concentration tested. (*b*) *T. urticae* adult females were infected with *B. megaterium* at ODs 0.1, 1 and 10, with LB and *E. coli* at OD 0.1 as controls. A reduction in the survival of *T. urticae* was observed after infection with *B. megaterium* with the two highest concentrations tested, but not for OD 0.1. In (*a*,*b*), vertical bars correspond to the standard errors of survival estimates, obtained from the Cox proportional hazards models. (*c*) Hazard ratios between *T. urticae* adults infected with LB or with bacteria (light grey, *B. megaterium*; dark grey, *E. coli*). Vertical bars correspond to the 95% CIs of the estimated hazard ratios. ***p < 0.001.

We tested survival of *T. urticae* following infection with *Escherichia coli*, a Gram-negative bacterium, or the Gram-positive *Bacillus megaterium*. Injecting spider mites with *E. coli* at three different concentrations—ODs 0.1, 1 or 10—significantly affected survival (Cox model, bacterial concentration effect, 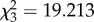, *p* = 0.0002; figure 1*a*). A pairwise comparison of the hazard ratios between spider mites injected with *E. coli* or with Luria broth (LB) confirmed that spider mite survival was severely affected (OD 0.1: *z* = 9.828, *p* < 0.0001; OD 1: *z* = 11.124, *p* < 0.0001 and OD 10: *z* = 14.267, *p* < 0.0001; figure 1*c*).

Injecting spider mites with *B. megaterium* at three different concentrations (ODs 0.1, 1 or 10) also significantly affected survival (Cox model, bacterial concentration effect, 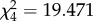; *p* < 0.0006; figure 1*b*). Hazard ratios revealed no significant change in survival between mites injected with LB or with *B. megaterium* at OD 0.1 (*z* = 1.769; *p* = 0.0769), but survival of *T. urticae* decreased significantly relative to the LB control at OD 1 and OD 10 (*B. megaterium* OD 1: *z* = 8.792, *p* < 0.0001; *B. megaterium* OD 10: *z* = 8.797, *p* < 0.0001; figure 1*c*).

The high mortality rate in *T. urticae* caused by infection with bacteria known to be non-pathogenic to *Drosophila melanogaster* [21] raised the possibility that our bacterial strains had an unexpected level of pathogenicity. To test this, we infected *D. melanogaster* adult females with the same bacteria and at the same concentrations applied to *T. urticae*. As previously reported [22,23], within the same time frame and bacteria inoculum range as our experiment with *T. urticae*, the survival of *D. melanogaster* was not reduced upon infection with either bacterium (Cox model, bacterial concentration effect, 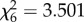, *p* = 0.7439; electronic supplementary material, figure S1).

### (b) The transcriptomic profile of *Tetranychus urticae* is unaltered upon infection

Next, we analysed genome-wide gene-expression patterns to assess the effect of bacterial infection on spider mites using a qPCR-validated microarray set-up [24]. Differential transcript levels were determined in mites injected with *E. coli* or with *B. megaterium* relative to mites injected with LB. Expression levels were measured 3, 6 and 12 h post-injection. Only a limited number of genes showed significant differences in expression between mites injected with or without bacteria and displayed an inconsistent response to bacteria across the three time points (electronic supplementary material, table S2). Moreover, even though the 34 orthologues of immunity-related *Drosophila* genes identified in the *T. urticae* genome had multiple probes on the array, none of these showed significant differential expression upon bacterial infection (electronic supplementary material, table S2).

**Figure 2.**
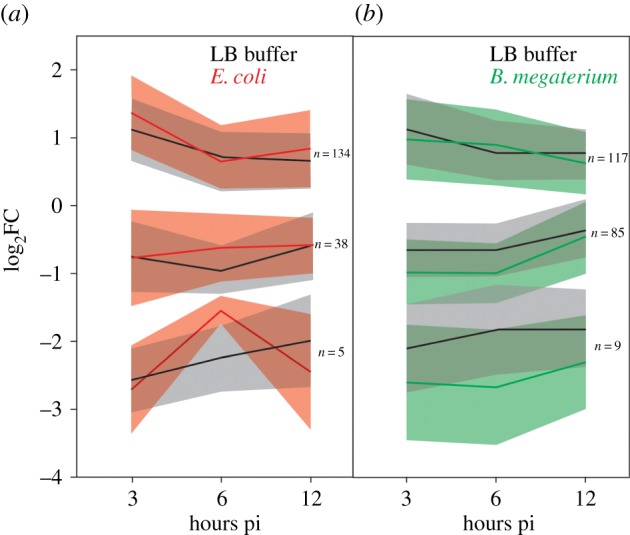
Spider mites do not trigger an induced immune response against bacterial infection. *T. urticae* adult females were infected with *E. coli*, *B. megaterium* and LB buffer by an injection method and collected 3, 6 or 12 h post-infection (pi) to analyse the transcriptomic responses. Using non-injected mites as a reference, the relative transcription levels of the gene sets that showed significant differential expression in any time point of each bacterial and LB-control treatment were subjected to hierarchical clustering based on the distance calculated by dynamic time warping alignments. Resulting clusters were grouped, of which the means (solid line) and confidence interval (*α*: 0.05) (shaded regions) are shown for infections with *E. coli* (in red) (*a*) or *B. megaterium* (in green) (*b*) together with their respective LB controls (in black).

By contrast, a more pronounced transcriptional response was observed between injected and non-injected mites. In these comparisons, we observed altered transcription of a total of 259 genes (out of 17 798 genes with probes on the array). More specifically, transcriptomic comparisons of the *E. coli* and *B. megaterium* injections, with their respective LB controls, uncovered a total of 177 and 211 differentially expressed genes, respectively, at any of the three time points (figure 2 and electronic supplementary material, figure S2). Only three genes were significantly differentially expressed in a consistent manner across every time point of every injection treatment (LB buffer, *E. coli* and *B. megaterium*). These are *tetur03g07900*, *tetur05g04720* and *tetur19g00860*, none of which shows any significant homology to known immunity genes (sequence data accessible at: http://bioinformatics.psb.ugent.be/orcae/overview/Tetur and http://www.uniprot.org/proteomes/ under UP000015104). No orthologues of *Drosophila* genes classified as immunity-related were present in any of these gene sets [19]. Of the 259 differentially expressed genes that showed significant differential expression in any comparison, 118 were given a Gene Ontology (GO) term by Blast2GO analysis [25]. Fisher's exact test showed that 16 and 12 GO terms were significantly over- and under-represented in the differentially expressed gene set, respectively (electronic supplementary material, table S3). No terms related to a physiological response to wounding were observed.

As indicated by the hierarchical clustering analysis based on time-series alignments of the relative transcription levels across the different time points (figure 2) and by corresponding gene-expression heatplots (electronic supplementary material, figure S2), the transcriptional response to injection did not appear to be time-dependent within the first 12 h. Indeed, no strong linear differential expression across the three time points was observed in any of the three injection treatments. Moreover, the lack of consistent differential expression across all time points of the different injection treatments indicates that the injection procedure itself did not elicit an immune response.

The transcriptional responses observed in individual comparisons do show that our pipeline is capable of identifying differential expression. Therefore, we interpret the lack of significantly distinct transcript levels in the direct comparisons of bacterial-injected versus LB-injected mites as caused by the virtual absence of immune response induction and not by a technical artefact.

### (c) *Sancassania berlesei* and *Tetranychus urticae* respond differently to systemic bacterial infection

To test whether the lack of an induced immune response in *T. urticae* is a general feature of the Acari, we mirrored the infections performed on the spider mite in the grain mite, *S. berlesei*.

**Figure 3.**
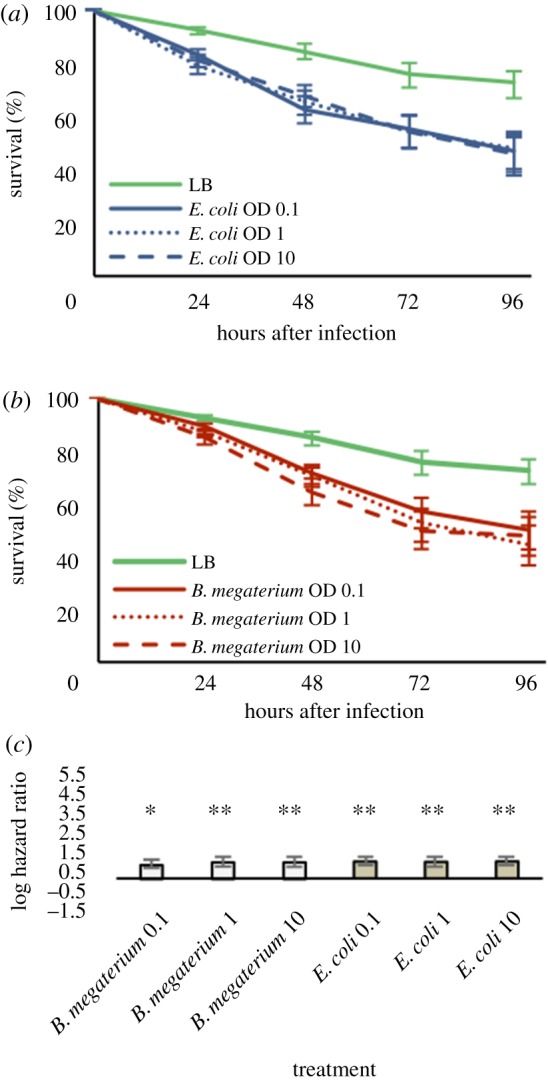
*Sancassania berlesei* can resist bacterial infection. (*a*,*b*) *S. berlesei* adult females were infected with *E. coli* (*a*) or *B. megaterium* (*b*) at three concentrations or with LB as control and their survival was followed daily over 4 days. Bacterial infection decreased survival but to a lesser degree than that observed in *T. urticae*. In (*a*,*b*), vertical bars correspond to the standard errors of survival estimates, obtained from the Cox proportional hazards models. (*c*) Hazard ratios of *S. berlesei* adults injected with bacteria relative to LB-injected controls. Vertical bars correspond to the 95% CIs of the estimated hazard ratios. *p < 0.05; **p < 0.01 and ***p < 0.001.

Overall, infection with bacteria decreased significantly the survival of *S. berlesei* (Cox model, bacterial concentration effect, 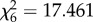, *p* = 0.0077; figure 3*a*,*b*). A reduction in survival was observed upon bacterial injection with either bacterial species at all tested concentrations (*E. coli* OD 0.1: *z* = 3.513, *p* < 0.0022; *E. coli* OD 1: *z* = 3.446, *p* = 0.0022; *E. coli* OD 10: *z* = 3.559, *p* = 0.0022; *B. megaterium* OD 0.1: *z* = 2.899, *p* = 0.0037; *B. megaterium* OD 1: *z* = 3.495, *p* = 0.0022; *B. megaterium* OD 10: *z* = 3.414, *p* = 0.0022; figure 3*c*).

However, when survival of *S. berlesei* and *T. urticae* is contrasted, it is unequivocal that the grain mite is far more capable of surviving infection than the spider mite (compare figure 1*a* with 3*a* and figure 1*b* with 3*b*). Finally, the same experiment using heat-killed bacteria did not show a differential survival of the two mite species (electronic supplementary material, figure S3). This observation discards the possibility of host inflammatory misregulation causing self-damage independently of bacterial action [26,27].

### (d) Bacterial proliferation correlates with host survival

**Figure 4.**
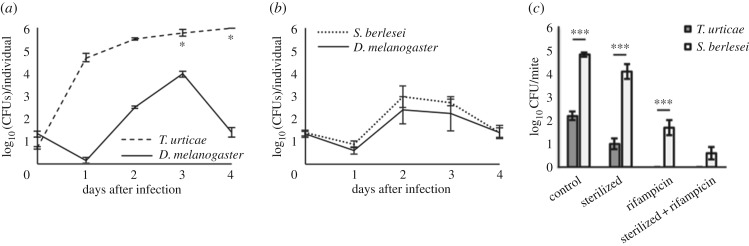
*Escherichia coli* proliferation in different species correlates with host survival patterns and gut microbiota size. *T. urticae*, *D. melanogaster* and *S. berlesei* were infected with 5–100 CFUs of *E. coli* per individual and bacterial dynamics within the host was quantified daily for up to 4 days after infection. (*a*) In *T. urticae*, an increase in the number of CFUs in living individuals was observed over the course of 2 days. At 3 and 4 days (asterisk), only dead individuals were plated as there were no survivors, but the CFUs did not increase significantly. (*b*) For *S. berlesei* injection*,* the dynamics of *E. coli* growth was similar to that of *D. melanogaster*. (*c*) Gut microbiota is virtually absent in *T. urticae* as opposed to *S. berlesei.* Untreated mites (control) or treated with different protocols to remove bacteria from their surface (sterilized), from their gut (rifampicin) and from both their surface and gut (sterilized and rifampicin) were homogenized and plated on a Petri dish containing LB agar. *T. urticae* is colonized by two to three orders of magnitude less bacteria than *S. berlesei*. ***p < 0.001.

We observed the dynamics of the bacterial inoculum after infection of both mite species and *D. melanogaster* by counting the number of colony-forming units (CFUs), over 4 days (figure 4).

We found significant differences in the number of CFUs between *T. urticae* and *D. melanogaster* (ANOVA, *F*_1,20_ = 134.066, *p* < 0.0001; figure 4*a*), across time points (ANOVA, *F*_4,20_ = 70.044, *p* < 0.0001), and in the interaction between time point and species (ANOVA, *F*_1,20_ = 25.131, *p* < 0.0001). The latter indicates that bacterial populations have distinct growth dynamics in *T. urticae* and in *D. melanogaster*. Indeed, in *T. urticae*, the number of CFUs increased across time points (figure 4*a*), whereas for *D. melanogaster* the number of CFUs started to decrease 2–3 days after injection, reaching in the last time point a value similar to the bacterial number initially injected (*t*_20_ = −0.201, *p* = 0.8427). By contrast, no significant differences were found between the dynamics of bacteria infecting *S. berlesei* and *D. melanogaster* (ANOVA, *F*_1,20_ = 0.51, *p* = 0.482; figure 4*b*), between time points (ANOVA, *F*_4,20_ = 2.287, *p* = 0.0957) and in the interaction between time points and species (ANOVA, *F*_4,20_ = 0.069, *p* = 0.9906).

This experiment provides a further line of evidence that *S. berlesei* is capable of fighting bacterial proliferation, contrary to *T. urticae*.

#### (e) *Tetranychus urticae* has an impoverished gut microbiota

We proceeded to quantify the microbiota in both mite species motivated by three important facts: (i) the ecological similarities between *T. urticae* and the aphid, (ii) the genomic evidence for the absence of the *Imd* gene and a general degeneration of the Imd pathway in the spider mite [13,14,19] and (iii) the central role of this pathway in gut homeostasis regarding regulation of the microbiota [28,29]. We quantified bacteria in individuals that were (i) surface-sterilized in bleach and alcohol, which should not have bacteria in their external surface; (ii) fed on rifampicin, which should not have bacteria in the gut or (iii) both, which should have neither and (iv) mites taken directly from their natural substrate, which should present both internally and externally associated bacteria. We homogenized individual adult females of *T. urticae* and *S. berlesei* from each treatment and plated them on LB agar plates (figure 4*c*) or extracted DNA to perform semi-quantitative PCR for the 16S gene (electronic supplementary material, figure S4). We found that the two mite species harbour a significantly different number of bacteria capable of growing on LB agar plates (ANOVA, mite species effect, *F*_1,72_ = 169.855, *p* < 0.0001) and that the treatments applied significantly decreased the number of bacteria (ANOVA, treatment effect, *F*_3,72_ = 93.543, *p* < 0.0001; figure 4*c*). We found a significant species by treatment interaction (ANOVA, *F*_3,72_ = 13.029, *p* < 0.0001), and the treatment with external sterilization and antibiotic treatment brings bacteria numbers to non-significantly different levels in both species (*t*_72_ = 1.924, *p* = 0.058). All other comparisons between treatments across species are highly significant (*t*_72_ > 5.492, *p* < 0.0001). It is particularly striking that, once sterilized and only harbouring the bacteria inside the gut, single crushed *T. urticae* individuals only generate around 10 CFUs, a difference of three orders of magnitude relative to their *S. berlesei* counterparts (figure 4*c*).

It is expected that an undetermined number of bacterial species will not be detected with the specific culture conditions used in this test. However, it is unlikely that, between the two mite species, the distribution of bacterial species, which can and cannot be grown in LB, will be significantly different to change the qualitative conclusion we reach.

A semi-quantitative PCR provided independent confirmation that *S. berlesei* has a microbiota in the order of one thousand times higher than that of *T. urticae.* Whereas amplification products are clearly visible using gDNA from sterilized *S. berlesei* after 25 cycles, only after 35 cycles are bands detectable from sterilized *T. urticae* (electronic supplementary material, figure S4). For example, contrasting bands from the pool of 100 non-sterilized *T. urticae* females (TP) at 35 cycles to the pool of 50 non-sterilized *S. berlesei* females (SP) at 25 cycles show comparable amplification products in the two bacterial types (2^10^ = 1024). Despite the poor quantitative power of this technique, its qualitative interpretation provides a rough estimate of the difference in bacteria present in either species, namely in their digestive tracts. Importantly, this difference is hardly attributable to any of the four most commonly described endosymbionts of spider mites [30], which are absent from our tested populations (electronic supplementary material, figure S5).

Together, these results concur in that most bacteria found in these species are inside the mite gut and that between *T. urticae* and *S. berlesei* their numbers differ by roughly three orders of magnitude.

## 3. Discussion

### (a) Spider mites are susceptible to bacterial infections

Using bacteria that are non-pathogenic to *D. melanogaster*, we have shown that spider mites infected over a 100-fold concentration range with both Gram-positive and Gram-negative bacteria display high mortality when compared with controls (both mock-infected and infected with heat-killed bacteria). In addition, no qualitatively different transcriptional change is induced consistently by the presence of bacteria. This is in sharp contrast with *Drosophila* which displays a strong upregulation of Imd and/or Toll pathways upon bacterial infection [3,31,32]. Although individual transcriptomic comparisons between injected and non-injected mites reveal differential expression, no consistent response to injection was observed across all time points and treatments. Therefore, wounding itself does not seem to induce an immune response. This is supported by the absence of enriched GO terms related to wound response in lumped individual transcriptomic responses (electronic supplementary material, table S3). Finally, in *T. urticae*, bacterial proliferation is maintained steadily across 4 days post-injection in consonance with its mortality profile. This strongly indicates that no resistance or tolerance mechanisms are operating in the spider mite and that uncontrolled bacterial proliferation caused the observed mortality rates.

Our data are consistent with the absence of an induced immune response but does not address the putative role of other constitutive defences involving expression of effectors, such as lysozymes, AMPs and ROS, or cellular immunity and phagocytosis [33]. Be that as it may, we show that these other candidate mechanisms in *T. urticae* are clearly insufficient in face of bacterial infections that are innocuous to *Drosophila* and other chelicerates such as ticks [34] or the wet grain mite *S. berlesei*, which occupies a very different ecological niche, namely bird litter and other substrates prone to bacterial proliferation and infection [20].

Bacterial infection affected the survival of *S. berlesei* but to a much lesser extent than that observed for *T. urticae*. Moreover, unlike *T. urticae*, *S. berlesei* was capable of controlling and reducing the bacterial load, and also mimicking the characterized immune response in *D. melanogaster* [35,36]. The observation that bacterial load in *S. berlesei* increases initially and decreases over time suggests that this mite species mounts an immune response against bacterial infection. The nature of this response in *S. berlesei*, induced and/or constitutive, remains to be determined. An induced response is supported in other chelicerates such as horseshoe crabs and spiders, which upregulate AMPs upon bacterial challenge [17,37], possibly without resorting to Imd or Toll pathways, as these are (at least partially) degenerated in genome-sequenced species [13,14]. In addition, anti-bacterial response may rely on higher basal levels of immune effectors such as circulating haemocyanin, C-reactive proteins and α2-macroglobulin deployed upon infection [38].

### (b) Life history correlates with immune degeneration in spider mites

Genomic and physiological studies of the pea aphid *A. pisum* uncovered a very similar pattern to our results on *T. urticae* [9,10,39,40]. Similarly to *T. urticae*, the genome of *A. pisum* misses important genes of the Imd pathway and several other *Drosophila* immune genes [9]. Infected aphids, in which lysozyme activity could be detected, did not upregulate AMPs, suggesting that aphids do not deploy an induced immune response upon bacterial challenge [9,10]. Instead, pea aphids seem to rely on another layer of defence, that is provided by their endosymbionts, namely *Regiella insecticola*, *Rickettsia* and *Spiroplasma*, to fight some fungal infections [41,42] and *Hamiltonella defensa* against parasitoid attack [43]. Possibly, endosymbionts commonly detected in *T. urticae* populations might also confer protection to attacks by fungi and other natural enemies [30] but, to our knowledge, there has been no report of endosymbiont protection against bacterial infections in arthropods (reviewed in [44]). In addition, infection with *E. coli* also induces significant mortality in the pea aphid [39]. In any case, by using bacteria-challenged spider mites devoid of four common endosymbionts, we aimed to specifically test the response on the host genomic and physiological levels [30].

As shown for the pea aphid [9,11,12], the degenerated immune genetic repertoire and immune response in *T. urticae* is also a secondary loss. One hypothesis for the genomic and physiological patterns observed in pea aphid immunity is that the virtually aseptic phloem diet of aphids would relax selection for the maintenance of costly immune response mechanisms [45]. This hypothesis may be extended to *T. urticae* as this species also feeds on a seemingly bacterial-free resource, the cytoplasmic content of leaf cells and phloem. This ecological scenario is opposite to that of the house fly, where the genomic expansion of immune-related genes may underlie its adaptation to septic environments [46]. Moreover, the shared degeneration of the Imd pathway in the spider mite and aphid reinforces this notion because of the central role played by this pathway in mediating the epithelial response to bacterial contacts in the gut and in the modulation of its bacterial contents [31,47–49]. Recent work has shown that, in addition to aphids, other phloem-sap feeders, such as white flies and psyllids, carry a reduced gut microbiota in both laboratory and natural populations [50]. Unfortunately, no information is yet available regarding immune responses in these insects.

The aseptic nature of the feeding source of spider mites is supported by the rough comparative characterization of the gut bacteria present in the two mite species studied here, which differs by several orders of magnitude. This ecological feature, by eliminating a constant necessity for balancing bacterial interactions (commensal or pathogenic), may relax the pressure to evolve or maintain a transcriptionally induced and regulated response. Further studies looking into immunity in other arthropods with obligate endosymbionts and/or with comparable dietary regimes will provide clearer answers about the forces driving convergent degeneration of this type of immune response.

## 4. Material and methods

(For most sections below, more detail is provided in electronic supplementary materials and methods.)

### (a) Arthropod and bacterial strains

#### (i) Tetranychus urticae

All spider mites used in this work are of the London strain, a reference line originally collected in Ontario, Canada. The line used for the Spider Mite Genome Sequencing project [19] was derived from this population. Spider mites were reared under laboratory conditions (25°C, 60% humidity and 16 L : 8 D photoperiod).

#### (ii) Sancassania berlesei

Grain mites (a kind gift from J. Radwan) were maintained in large numbers in Petri dishes (6 cm diameter) with fly food under laboratory conditions (25°C, 50% humidity and 12 L : 12 D photoperiod).

#### (iii) Drosophila melanogaster

The w^1118^ laboratory stock kept under standard laboratory conditions was used in the survival assays and the dynamics of bacterial infection.

#### (iv) Bacteria

*Escherichia coli* (Gram −) and *B. megaterium* (Gram +) stocks were kept at −80°C and bacteria were plated onto Petri dishes with LB. Per experiment, one colony was picked from selective medium cultures, transferred to liquid LB and grown overnight at 37°C for *E. coli* and at 30°C for *B. megaterium*.

### (b) Survival assays

*Tetranychus urticae*, *S. berlesei* and *D. melanogaster* survival was monitored after injection with *E. coli* or *B. megaterium* for up to 96 h at 24-h intervals. Individuals were injected with LB with or without bacteria. For the former treatment, we used three different concentrations of bacteria, OD 0.1, OD 1 and OD 10 measured with a BioRad SmartSpec 3000. OD 10 corresponds to 5 × 10^9^ cell ml^−1^; OD 1 and OD 0.10 were obtained by diluting bacteria at OD 10 in LB at a 1 : 10 ratio and 1 : 100 ratio, respectively.

### (c) *Tetranychus urticae* transcriptome analysis

Female adult spider mites were injected with *E. coli* or *B. megaterium* at OD 1 concentration with LB as a negative control, or were left unmanipulated. Three, 6 and 12 h post-infection, four biological replicates of every injection treatment were collected. Two biological replicates were collected from non-treated, non-injected mites. Every RNA sample was extracted from a pool of 300 mites and labelled with Cy5 or with Cy3. Significant differential expression was identified by an empirical Bayes approach employing cut-offs for the Benjamini–Hochberg FDR adjusted *p*-values and log_2_-converted fold change at 0.05 and 1, respectively [51]. The proxy, NbClust and dtw packages in R were used in the distance matrix construction and clustering of the transcriptomic responses. Distance measures were generated through alignments of the relative transcription levels (injected versus non-injected) using dynamic time warp algorithms. This technique allows for the comparison of the transcriptomic responses over time [52,53].

### (d) Infection with heat-killed bacteria

*Tetranychus urticae* and *S. berlesei* survival was measured after infection (injection or pricking, respectively) with live or heat-killed *E. coli* or *B. megaterium* at OD 10*.* Three replicates of 30, 1- to 3-day-old, adult females were used per treatment: LB, live *E. coli*, heat-killed *E. coli*, live *B. megaterium* and heat-killed *B. megaterium*. Survival was monitored every 24 h over 4 days.

### (e) Dynamics of bacterial growth

We infected 150 females of *T. urticae*, *S. berlesei* and *D. melanogaster* with 5–100 CFUs of *E. coli* per individual. The dynamics of the bacterial population was followed every 24 h from 0 to 96 h. At each time point, three replicates of four individuals were homogenized in 50 µl of LB and serially diluted. Homogenates (4 µl) were plated in triplicate on LB plates supplemented with 100 mg ml^−1^ kanamycin and incubated overnight. The next day, the number of CFUs was counted. For *S. berlesei* and *D. melanogaster*, only the homogenates of the individuals alive were plated. However, for *T. urticae*, individuals collected and plated at 72 and 96 h were dead because no survivors could be recovered at those time-points. The standard error was obtained by dividing the standard deviation of the three biological replicates datapoints (values in log_10_ CFU), divided by the square root of three, the number of samples for each time point/species.

### (f) Estimating the microbiota associated with each mite species

#### (i) Sterilization and rifampicin treatments

To measure the approximate number of bacteria present either outside or inside each mite species, adult *T. urticae* and *S. berlesei* females were exposed to one of four different treatments: no sterilization, sterilization, feeding on rifampicin and feeding on rifampicin plus sterilization. Single individuals were homogenized in 50 µl of LB, plated (4 µl) in LB agar and incubated overnight at 30°C. The next day, the number of CFUs was counted and used as a proxy to estimate the microbiota associated with each mite (five per treatment and per species).

#### (ii) Semi-quantitative PCR of bacterial 16S

DNA was extracted from individual mites after sterilization (GenElute™ Mammalian Genomic DNA Miniprep Kit, Sigma–Aldrich Co., St Louis, USA). PCRs were performed using standardized concentration of DNA templates (around 4.5 ng µl^−1^) and using universal primers for the bacterial 16S gene defined by Lane [54], 27f: GAG AGT TTG ATC CTG GCT CAG and 1495r: CTA CGG CTA CCT TGT TAC GA. PCR amplification conditions were as follows: 15 min at 95°C, followed by 15/20/25/30 three-step cycles of 94°C for 30 s, 58°C for 1 min 30 s, 72°C for 1 min followed by a final step of 10 min at 72°C.

### (g) Statistical analysis

Analyses were carried out using the R statistical package (v. 3.1.2). To compare survival between uninfected and infected individuals, we used Cox proportional hazards mixed-effect models (*coxme*, coxme package). To compare the dynamics of bacterial infection, a linear model on log_10-_transformed bacterial counts was employed with species, time point and the interaction between species and time point as fixed factors. To analyse the microbial community of both mite species, a linear model on the log_10_-transformed bacteria counts was employed with species and treatment as fixed factors. In both tests, the significance of the explanatory variables was tested using type I ANOVA. Pairwise comparisons between time points or treatments were performed using the *lsmeans* function of the package lsmeans. When a significant interaction was found, comparisons were done separately for each species or each treatment.

## Supplementary Material

Matos,Wybouw,Martins_suppF&T

## Supplementary Material

Suppl_Materials and Methods

## Data accessibility

*Tetranychus urticae* sequence data may be accessed at: http://bioinformatics.psb.ugent.be/orcae/overview/Tetur and http://www.uniprot.org/proteomes/ under UP000015104. Transcriptomic data may be accessed at the GEO database of NCBI under accession number GSE64199.
