# Supplementary material for: Tetranychus urticae mites do not mount an induced immune response against bacteria
Source: Proc Biol Sci. 2017 Jun 7;284(1856):20170401. doi: 10.1098/rspb.2017.0401 (PMC5474072; doi:10.1098/rspb.2017.0401)
Supplement: Matos,Wybouw,Martins_suppF&T [file rspb20170401supp1.pdf]

Supplementary Figures and Tables

Supplementary Figure 1

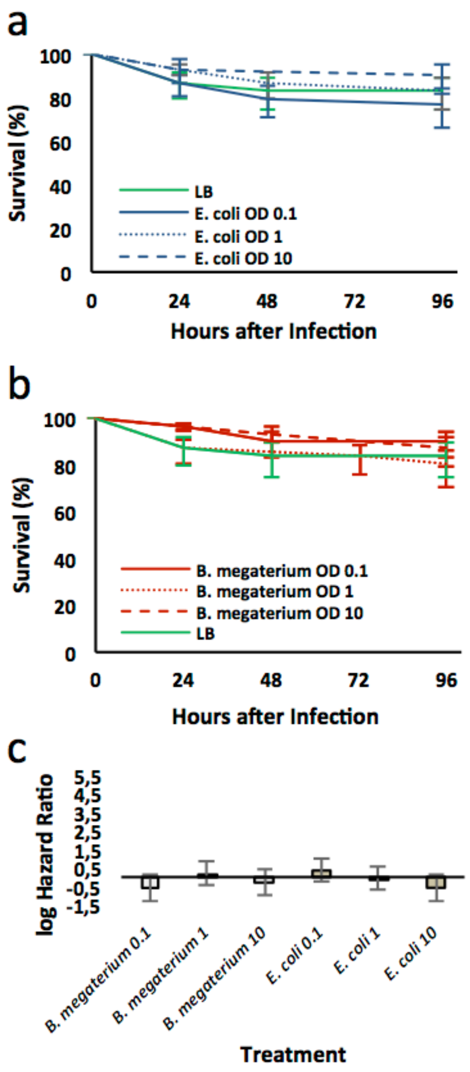

Supplementary Figure 1 – *D. melanogaster* survival is not affected by infection with either *E. coli* or *B. megaterium*

D. melanogaster was infected with *E. coli* (a) *or B. megaterium* (b) at three different concentrations and LB as control. Survival of *D. melanogaster* is not reduced upon bacterial infection. In a) and b), vertical bars correspond to the standard errors of survival estimates, obtained from the Cox proportional hazards models. In (c), Hazard ratios of *D. melanogaster* adults injected with bacteria relative to LB injected controls. Vertical bars correspond to the 95% confidence intervals of the estimated hazard ratios.

Supplementary Figure 2

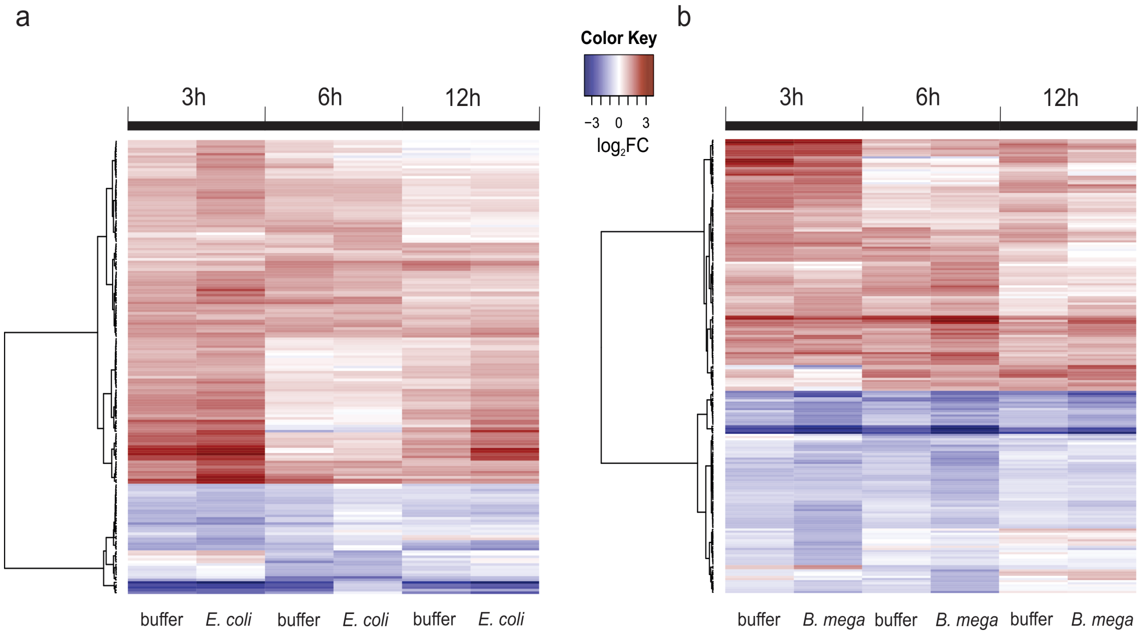

Supplementary Figure 2 - Bacterial infection does not trigger differential gene expression in *T. urticae*. Gene-expression heatplots of genes differentially expressed upon injection of *E. coli* (a) and *B. megaterium* (b), with their respective LB-controls, in any of the three time points ($log_2FC>1$ and FDR-corrected p-value<0.05). Genes (a: n=177 and b: n=211) were hierarchically clustered (Euclidean, ward) based on their relative expression to non-injected controls.

Supplementary Figure 3

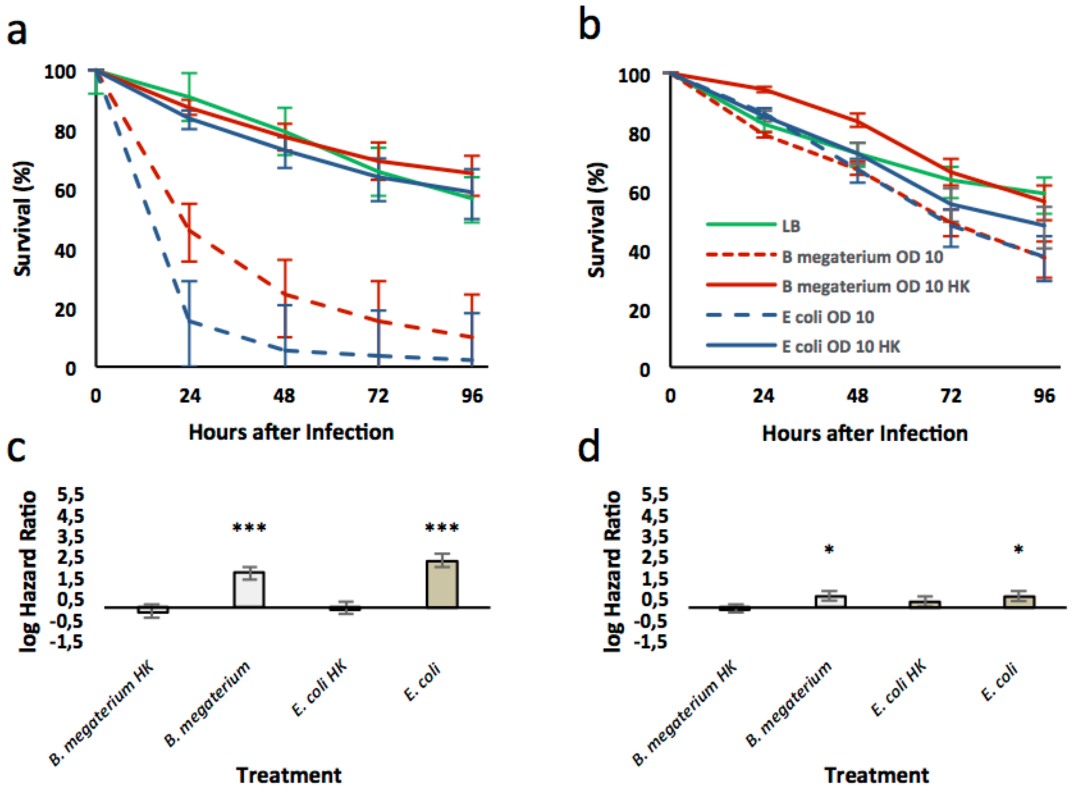

Supplementary Figure 3 – Infection with heat-killed bacteria did not reduce the survival of mites

Mites were infected with an OD 10 of live or dead bacteria (HK-heat killed), *E. coli* or *B. megaterium*. Heat-killed bacteria did not reduce survival of *T. urticae* (a) nor *S. berlesei* (b). In a) and b), vertical bars correspond to the standard errors of survival estimates, obtained from the Cox proportional hazards models. Panels c) and d) show the Hazard ratios for the treatments presented in (a) and (b), respectively, with vertical bars correspond to the 95% confidence intervals. *P<0,05; **P<0,01 and ***P< 0,001.

Supplementary Figure 4

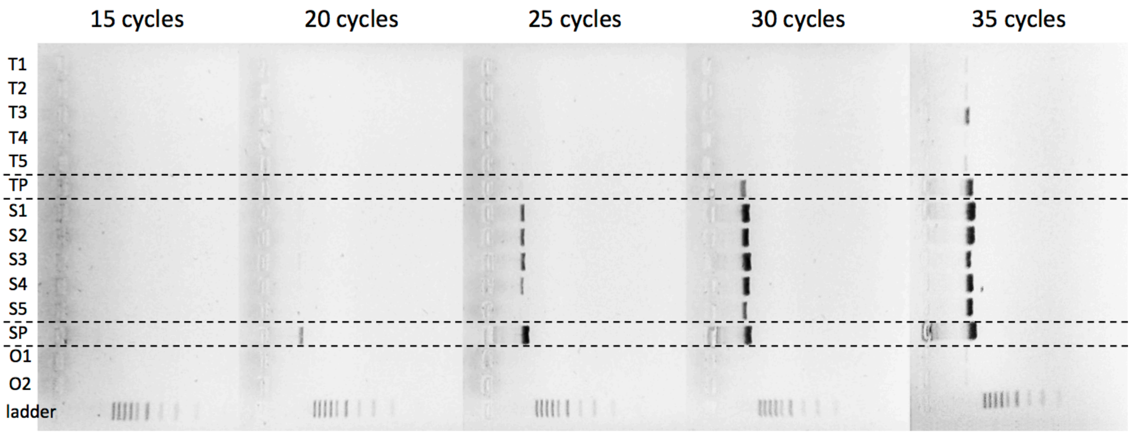

Supplementary Figure 4 – Semi-quantitative PCR showing that *T. urticae* have a severely reduced gut microbiota relative to *S. berlesei*.

The comparison between TP and SP suggests a difference in the order of three orders of magnitude in accordance to the plating experiment shown above. T1-5: Single sterilized *T. urticae* females; TP: Non-sterilized *T. urticae* pool of 100 females; S1-5: Single sterilized *S. berlesei* females; SP: Non-sterilized *S. berlesei* pool of 50 females; O1: DNA extraction negative control; O2: PCR negative control.

Supplementary Figure 5

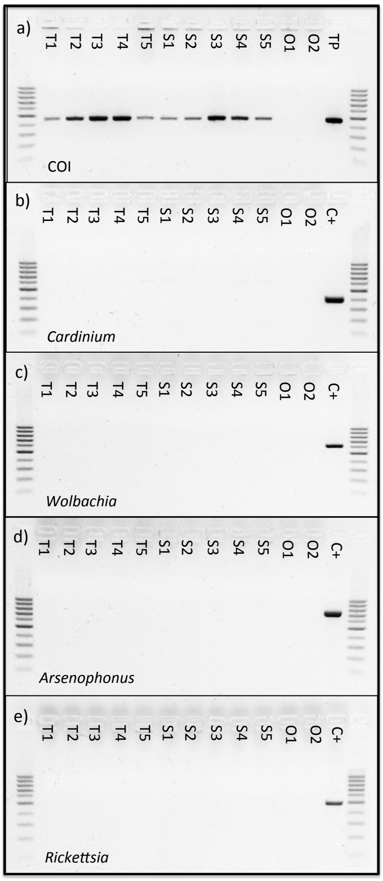

Supplementary Figure 5 – *S. berlesei* and *T. urticae* populations are free of common endosymbionts

Both mite species, *T. urticae* and *S. berlesei*, are free of common endosymbionts as shown by the absence of amplification using standard primers. Diagnostic PCR for four reported mite endosymbionts was performed on 5 individuals of each species, T1-5, are sterilized *T. urticae* single females and, S1-5, are sterilized *S. berlesei* single females. As positive controls, we used gDNA from infected mites for each of the tested endosymbionts. (a) Control PCR using cytochrome oxidase primers COI-F: TGA TTT TTT GGT CAC CCA GAA G and COI-R: TAC AGC TCC TAT AGA TAA AAC, as described in Navajas *et al* [1]. (b) PCR for the 16S rRNA gene of *Cardinium* (and other related Bacteroidetes symbionts) using primers ChF: TAC TGT AAG AAT AAG CAC CGG C and ChR: GTG GAT CAC TTA ACG CTT TCG, as reported in Zchori-Fein & Perlman [2]. (c) The *Wolbachia* gene *wsp* was amplified with primers wsp81F: TGG TCC AAT AAG TGA TGA AGA AAC and wsp691R: AAA AAT TAA ACG CTA CTC CA[3]. (d) *Arsenophonus* yaeT gene was amplified using primers from Duron et al [4], yaeTF: GCA TAC GGT TCA GAC GGG TTT G and yaeTR: GCC GAA ACG CCT TCA GAA AAG. (e) *Rickettsia prowazekii* gltA gene was amplified with primers described by Davis and colleagues [5], RICS741F: CAT CCG GAG CTA ATG GTT TTG C and RCIT1197R: CAT TTC TTT CCA TTG TGC CAT C.

As a preliminary experiment using PCR, we tested our populations for the presence of four of the most common endosymbionts of arthropods, namely Wolbachia, Rickettsia, Cardinium and Arsenophonus (Suppl. Fig. 4). The absence of these symbionts suggests that bacterial growth on plates and 16S

amplification can be ascribed to by bacteria on the surface of the mite or inside the gut as part of the microbiota. Our four treatments consisted of individuals that were 1) surface-sterilized in bleach and alcohol, which should not have bacteria in their external surface; 2) fed on rifampicin, which should not have bacteria in the gut; or 3) both, which should have neither and 4) mites taken directly from their natural substrate, which should present both internally and externally associated bacteria.

Supplementary Table 1

Gene	Gene function/ Pathway	*D. melanogaster*	*T. urticae*
PGRPs	Recognition	13(a,c) or 7(b)	1
GNBPs	Recognition	3	0
Toll	TOLL	9	4
Spätzle	TOLL	6	5(b,c) or 6(a)
MyD88	TOLL	1	1
Pelle	TOLL	1	1(c) or 2(a,b)
Cactus	TOLL	1	1
Tube	TOLL	1	0(a,b,c) or 1(c)
Dorsal	TOLL	1	1
Dif	TOLL	1	0
Imd	IMD	1	0
dFADD	IMD	1	0
Dredd	IMD	1	0
TAK	IMD	1	1
Kenny	IMD	1	0
Relish	IMD	1	1
attacins	Effector/AMP	4	0
cecropins	Effector/AMP	4	0
defesin	Effector/AMP	1	0
diptericin	Effector/AMP	2	0
drosocin	Effector/AMP	1	0
drosomycin	Effector/AMP	6	0
metchnikowin	Effector/AMP	1	0
Lysozyme	Effector	13(b) or 7(c)	3

Supplementary Table 1 - A comparison of the presence and number of genes associated with bacterial innate immunity in the genome of *Drosophila melanogaster* and *T. urticae*. Data was collected from references a) Grbic et al 2011, *Nature [6];* b) Palmer and Jiggins 2015, *Mol. Biol. Evol.[7]*; c) Bechsgaard et al 2016, *JEB[8]***.** Note that in some cases different references indicate different number of genes.

Supplementary Table 2

DEGs upon *E. coli* infection

	3 h pi		6 h pi		12 h pi		
TeturID	log$_2$FC	adj. p-val	log$_2$FC	adj. p-val	log$_2$FC	adj. p-val	**Description**
tetur04g04350	1.50	7.82E-04	-1.05	1.80E-02	-	-	UDP-glycosyltransferase
tetur03g05190	1.00	8.37E-04	-	-	-	-	cytochrome P450 monooxygenase
tetur04g01060	1.07	2.53E-03	-	-	-	-	fatty acyl CoA reductases
tetur11g05570	1.26	2.29E-02	-	-	-	-	hypothetical protein
tetur03g08300	-	-	1.79	2.74E-03	-	-	hypothetical protein
tetur09g06230	-	-	1.20	1.89E-02	-	-	cuticle protein
tetur04g01580	-	-	1.67	2.38E-02	-	-	cuticle protein
tetur04g01610	-	-	1.78	4.33E-02	-	-	cuticle protein
tetur11g05230	-	-	-	-	1.26	2.04E-04	lipocalin
tetur11g05210	-	-	-	-	1.15	7.55E-04	lipocalin
tetur06g03070	-	-	-	-	1.01	6.14E-03	hypothetical protein
tetur06g03350	-	-	-	-	1.01	6.32E-03	lipocalin
tetur06g02780	-	-	-	-	1.30	1.37E-02	hypothetical protein
tetur16g03410	-	-	-	-	1.10	1.69E-02	lipocalin

DEGs upon *B. megaterium* infection

	3 h pi		6 h pi		12 h pi		
TeturID	log$_2$FC	adj. p-val	log$_2$FC	adj. p-val	log$_2$FC	adj. p-val	**Description**
tetur11g05210	-1.02	3.48E-03	-	-	-	-	lipocalin
tetur07g05940	-1.31	8.90E-03	-	-	-	-	intradiol ring-cleavage dioxygenase
tetur03g07450	-1.05	1.45E-02	-	-	-	-	PAN/APPLE-like domain proteins
tetur11g05730	-	-	-1.04	4.36E-03	-	-	hypothetical protein
tetur05g05030	-	-	-1.06	1.98E-02	-	-	UDP-glycosyltransferase
tetur02g14420	-	-	-	-	1.11	4.56E-06	hypothetical protein

tetur04g04350	-	-	-	-	1.10	1.12E-02	UDP-glycosyltransferase

Supplementary Table 2 - Differentially expressed *T. urticae* genes upon bacterial infection, using a LB-injection control.

Two types of bacteria were injected in adult female *T. urticae*, Gram-negative bacterium *E. coli* and a Gram-positive bacterium *B. megaterium*. Transcriptomic analysis was performed 3, 6 and 12 h after injection, using LB injected mites as control. The cut-offs for differential expression for log2FC and BH FDR-adjusted p-values were 1 and 0.05, respectively. *T. urticae* genes differentially expressed upon infection of both bacterial species are indicated in bold. TeturIDs are accessible at the ORCAE website (http://bioinformatics.psb.ugent.be/orcae). Gene descriptions are based on manual annotations or on conserved protein domains [7][8][6].

Supplementary Table 3

GO-ID	Term	Category	FDR	DEG	Genome	O/U
GO:0001953	negative regulation of cell-matrix adhesion	P	3.11E-02	3	9	O
GO:0001957	intramembranous ossification	P	1.68E-04	4	5	O
GO:0005506	iron ion binding	F	1.68E-04	14	177	O
GO:0005576	extracellular region	C	3.96E-02	11	231	O
GO:0005764	lysosome	C	1.40E-02	8	103	O
GO:0008234	cysteine-type peptidase activity	F	2.50E-03	10	130	O
GO:0009055	electron carrier activity	F	7.53E-03	10	151	O
GO:0015979	photosynthesis	P	8.93E-04	3	3	O
GO:0015995	chlorophyll biosynthetic process	P	8.93E-04	3	3	O
GO:0016117	carotenoid biosynthetic process	P	2.50E-03	3	4	O
GO:0016120	carotene biosynthetic process	P	8.93E-04	3	3	O
GO:0016166	phytoene dehydrogenase activity	F	3.11E-02	2	2	O
GO:0020037	heme binding	F	5.11E-03	10	143	O
GO:0031409	pigment binding	F	5.18E-04	5	15	O
GO:0045453	bone resorption	P	8.93E-04	4	8	O
GO:0055114	oxidation-reduction process	P	1.68E-04	25	576	O
GO:0003676	nucleic acid binding	F	1.05E-02	4	1360	U
GO:0005515	protein binding	F	1.68E-04	15	3041	U
GO:0006464	cellular protein modification process	P	1.56E-02	1	891	U
GO:0007275	multicellular organismal development	P	3.90E-02	5	1349	U
GO:0010467	gene expression	P	1.11E-03	3	1420	U
GO:0016070	RNA metabolic process	P	2.50E-03	2	1192	U
GO:0019219	regulation of nucleobase-containing compound metabolic process	P	4.10E-02	1	792	U
GO:0031981	nuclear lumen	C	2.88E-02	0	680	U
GO:0034645	cellular macromolecule biosynthetic process	P	1.24E-03	2	1267	U
GO:0043232	intracellular non-membrane-bounded organelle	C	4.62E-04	0	1029	U
GO:0043234	protein complex	C	9.14E-04	1	1144	U
GO:0060255	regulation of macromolecule metabolic process	P	3.11E-02	2	989	U

Supplementary Table 3 - **Enriched Gene Ontology categories of all *T. urticae* genes showing differential expression in injection *vs* non-injection treatments.**

Categories labelled with F, P and C relate to Molecular Function, Biological Process and cellular component, respectively. The FDR-corrected p-values are listed. DEG stands for differentially-expressed genes. The O/U column shows whether the GO-term was over or under represented in the differentially expressed gene set.

REFERENCES

1. M. Navajas, Gutierrez M, Lagnel J, Boursot P. 1996 Mitochondrial cytochrome oxidase I in tetranychid mites: a comparison between molecular phylogeny and changes of morphological and life history traits. *Bull Ent Res* **86**, 407–417.

2. Zchori-Fein E, Perlman SJ. 2004 Distribution of the bacterial symbiont Cardinium in arthropods. *Mol. Ecol.* **13**, 2009–2016.

3. Braig HR, Zhou W, Dobson SL, O'Neill SL. 1998 Cloning and Characterization of a Gene Encoding the Major Surface Protein of the Bacterial Endosymbiont *Wolbachia pipientis*. *Journal of Bacteriology* **180**, 2373–2378.

4. Duron O, Wilkes TE, Hurst GD. 2010 Interspecific transmission of a male-killing bacterium on an ecological timescale. *Ecol Lett* **13**, 1139–48.

5. Davis MJ, Ying Z, Brunner BR, Pantoja A, Ferwerda FH. 1998 Rickettsial relative associated with papaya bunchy top disease. *Curr. Microbiol.* **36**, 80–84.

6. Grbic M *et al.* 2011 The genome of *Tetranychus urticae* reveals herbivorous pest adaptations. *Nature* **479**, 487–92.

7. Palmer WJ, Jiggins FM. 2015 Comparative Genomics Reveals the Origins and Diversity of Arthropod Immune Systems. *Molecular Biology and Evolution* **32**, 2111–2129.

8. Bechsgaard J, Vanthournout B, Funch P, Vestbo S, Gibbs RA, Richards S, Sanggaard KW, Enghild JJ, Bilde T. 2016 Comparative genomic study of arachnid immune systems indicates loss of beta-1,3-glucanase-related proteins and the immune deficiency pathway. *Journal of Evolutionary Biology* **29**, 277–291.
