# Supplementary material for: Tetranychus urticae mites do not mount an induced immune response against bacteria
Source: Proc Biol Sci. 2017 Jun 7;284(1856):20170401. doi: 10.1098/rspb.2017.0401 (PMC5474072; doi:10.1098/rspb.2017.0401)
Supplement: Suppl_Materials and Methods [file rspb20170401supp2.pdf]

Supplementary Materials and Methods

Arthropod and bacterial strains:

Tetranychus urticae: Spider mites used in the survival assays and gene-expression microarray experiments were maintained at the University of Crete, Greece. For all other experiments, a population maintained at the University of Lisbon, Portugal was used. In both labs, spider mites were maintained on potted bean plants (*Phaseolus vulgaris*) in high census and cultured in a herbivore-free environment. Neither population was the object of antibiotic treatment prior to the experiments here described.

Mites were reared on three-week-old adult plants in large numbers, with overlapping generations, and transferred to a new plant every 10-15 days. Under laboratory the described laboratory conditions, these populations have a generation time of 10-12 days.

Sancassania berlesei: The population of grain mites used in this study was founded from 500 individuals derived from a population maintained at Jagiellonian University, Krakow, Poland (a kind gift from J. Radwan). At Instituto Gulbenkian de Ciência (IGC), Oeiras, Portugal, this population was maintained in 15 different petri dishes, with overlapping generations. Every other week, 40-50 adult females and 5-10 males from different dishes were mixed and transferred to a new dish with fresh food. Under lab conditions (25°C, 50% humidity and L:D 12:12 photoperiod), *S. berlesei* has a generation time of 12-14 days.

Drosophila melanogaster: The w^{1118} laboratory stock was maintained in bottles (10cm height and 4.5cm diameter) with standard fly food, with more than 50 individuals per bottle. Under lab conditions (25° C, 50% humidity and L:D 12:12 photoperiod), these flies have a generation time of 9-10 days.

Bacteria: The *E. coli* strain tested was DH5α carrying the plasmid pSCM21, which contains GFP and resistance to kanamycin markers. The *B. megaterium* strain, a generous gift from A. Henriques (ITQB), is naturally resistant to lyncomycin and was isolated from fecal material from organically reared broilers [1]. *B. megaterium* was grown at 30°C. Both bacteria were grown to the desired optical density (OD) determined by spectrophotometry.

Survival Assays

T. urticae: Two-day old adult female spider mites were injected in the hemocoel cavity with either LB or LB+bacteria using borosilicate glass capillaries GC1OOF-10 (Harvard Apparatus, Kent, UK) attached to a IM 3000 automatic microinjector with injection pulses of 0,4 seconds at 20-25 psi (pound per square inch) (“injection”). After injection with *E. coli* (sample sizes: LB: 108, OD 0.1: 111, OD1: 133 and OD10: 144), spider mites from each treatment were transferred to a single primary bean leaf (10 cm long and 7 cm width) placed on top of cotton wool in a Petri dish (29cm diameter). Survival was monitored at 24 and 48 hours after infection. For the experiment with *B. megaterium*, 120 spider mites were injected per treatment. After injection, 3 groups of 40 spider mites were transferred to clean bean leaves and survival was measured at 24, 48 and 96 hours after injection.

S. berlesei: 1 to 3-day adult females were infected by piercing the female’s abdomen with an insect needle dipped in either LB or LB+bacteria – *E. coli* or *B. megaterium* (“pricking”). We were unable to use the microinjector as the wound caused by the glass needle led to mortality rates above 50% in the LB control after 24 hours (data not shown). After pricking, mites were transferred in groups of 10 to Petri dishes (7cm diameter) filled with agar (1.5%) and a top layer of yeast. 3 blocks were performed for each treatment with 120 mites tested per block (30 per treatment). Survival was registered at 24h intervals during 96 hours. Note that the results concerning *E. coli* and

B. megaterium were split into panels 3a and 3b to ease visualization of effects of either bacterium, although the experiments were performed simultaneously.

S. berlesei is five times heavier than *T. urticae* but differences in the bacterial inocula span over a 100–fold range.

D. melanogaster: 1 to 3-day old adult females were pricked in the abdomen with an insect needle dipped in either LB or LB+bacteria – *E. coli* or *B. megaterium*. After pricking, flies were transferred to a tube containing fly food. Tests were performed with a total of 210 flies divided into groups of 30 per treatment. Survival was measured every 24 hours over a period of 96 hours.

***T. urticae* transcriptome analysis**

Gene expression micro-Array

Cyanine labelled cRNA (generated using Low Input Quick Amp Labeling Kit, Agilent Technologies) was pooled and hybridized to an Agilent custom Sureprint GE 8X60k array following the Gene Expression Hybridization Kit (Agilent Technologies). The custom array (GEO platform GPL16890) holds gene-specific probes for 17,798 genes and covers 93.16% of all coding sequences within the mite genome. The platform has been used and validated by qPCR in a number of previous studies. As in other studies [2][3][4], slides of were washed and scanned using the Gene Expression Wash Buffer Kit (Agilent Technologies) and an Agilent High-Resolution Microarray Scanner (Agilent Technologies), respectively. Image output files were retrieved by Agilent Feature Extraction software (Protocol GE2_107_Sep09) and transferred to limma package (Linear Models for Microarray and RNA-seq Data) for final statistical processing [5]. Array background intensities were corrected by the "normexp"-method using an offset

of 50 [6]. Data was subsequently normalized both within and between arrays ("loess"- and "Aquantile"-method, respectively). Quality of the final, processed data was evaluated using the arrayQualityMetrics [7]. In the linear model of the data, intra-spot correlations were incorporated [8].

Gene-expression data is accessible on the GEO platform of NCBI under number GSE64199 as "Transcriptomic responses in the spider mite *Tetranychus urticae* to bacterial infection".

Assignment of GO-terms

Assignment of GO-terms was performed using Blast2GO software [9]. An E-value cut-off of $1e^{-15}$ was used for BLAST analysis. Annotation was performed with a cut-off of $1e^{-15}$ and further updated by InterPro. A two-tailed Fisher's exact test identified significantly enriched GO-terms amongst differentially expressed genes using the full *T. urticae* genome as reference (Benjamini-Hochberg FDR corrected p-value< 0.05). The resulting number of GO-terms was reduced to the most specific terms.

Infection with heat-killed bacteria

E. coli or *B. megaterium* at OD10 were killed by placing an Eppendorf tube containing 0.5 ml at 80°C, for 1 hour. To confirm if bacteria were killed, we checked for absence of colony-forming units (CFUs) on plates incubated overnight in which 4µl of these bacteria cultures had been plated at 15-minute intervals (data not shown).

Dynamics of bacterial growth

To compare *T. urticae* and *D. melanogaster*, 150 adult females of each species were infected by injection (*T. urticae*) or pricking (*D. melanogaster*). For *S. berlesei*, we infected 150 *S. berlesei* adult females and another 150 adult *D. melanogaster* females, using an insect needle. After infection, individuals were placed on clean bean leaves (*T. urticae*) in petri dishes with 1.5% agar and a layer of yeast (*S. berlesei*) or in tubes with fly food (*D. melanogaster*). At each time point, three replicates of 4 individuals were homogenized in 50µl of LB and serially diluted. Homogenates (4 µl) were plated in triplicate on LB plates supplemented with 100 mg/ml kanamycin and incubated overnight. The next day, the number of CFUs was counted.

Estimating the microbiota associated to each mite species

<u>Sterilization and rifampicin treatments</u>

The sterilization procedure consisted of sequential passages through ethanol 70% (3x) and a final rinse in water for 1 minute. For the rifampicin treatment of *T. urticae*, spider mites were placed on a bean leaf on top of cotton inside a petri dish containing water with 0.1 w/v of antibiotic. For *S. berlesei*, mites were placed in a petri dish with fly food with rifampicin (0.1% w/v). In both cases, mites were kept in the dishes for 48 hours.

Statistical analysis

In the survival experiments, individuals that escaped from the petri dishes or drowned during the 4-day period were counted as censored observations. In the experiments testing survival of *T. urticae*, *S. berlesei* and *D. melanogaster* after injection, the model

included LB (OD 0) and the different bacterial doses as a fixed factor and replicates nested into treatment as a random factor. For the experiments regarding the survival after injection of heat-killed bacteria, the model included treatment (live or heat-killed bacteria) as fixed factor and replicates nested into treatment and treatment nested into block as random factors. The significance of the explanatory variables was tested by comparing the likelihood ratio (approximately distributed as a chi-squared distribution[10]) between the full model and a null model with only random effects [11]. When significant, hazard ratios (HR) were obtained from these models as an estimate of death rate differences between LB and the other fixed factor levels (the reported bacterial species/doses/treatment combinations).

References:

1. Barbosa, T. M., Serra, C. R., La Ragione, R. M., Woodward, M. J. & Henriques, A. O. 2005 Screening for bacillus isolates in the broiler gastrointestinal tract. *Appl. Environ. Microbiol.* **71**, 968–978. (doi:10.1128/AEM.71.2.968-978.2005)

2. Dermauw, W., Wybouw, N., Rombauts, S., Menten, B., Vontas, J., Grbic, M., Clark, R. M., Feyereisen, R. & Van Leeuwen, T. 2013 A link between host plant adaptation and pesticide resistance in the polyphagous spider mite Tetranychus urticae. *Proc. Natl. Acad. Sci. U.S.A.* **110**, E113–122. (doi:10.1073/pnas.1213214110)

3. Bryon, A., Wybouw, N., Dermauw, W., Tirry, L. & Van Leeuwen, T. 2013 Genome wide gene-expression analysis of facultative reproductive diapause in the two-spotted spider mite Tetranychus urticae. *BMC Genomics* **14**, 815. (doi:10.1186/1471-2164-14-815)

4. Khalighi, M., Dermauw, W., Wybouw, N., Bajda, S., Osakabe, M., Tirry, L. & Van Leeuwen, T. 2016 Molecular analysis of cyenopyrafen resistance in the two-spotted spider mite *Tetranychus urticae*: Molecular analysis of cyenopyrafen resistance in *T. urticae*. *Pest Management Science* **72**, 103–112. (doi:10.1002/ps.4071)

5. Smyth, G. K. 2004 Linear Models and Empirical Bayes Methods for Assessing Differential Expression in Microarray Experiments. *Statistical Applications in Genetics and Molecular Biology* **3**, 1–25. (doi:10.2202/1544-6115.1027)

6. Ritchie, M. E., Silver, J., Oshlack, A., Holmes, M., Diyagama, D., Holloway, A. & Smyth, G. K. 2007 A comparison of background correction methods for two-colour microarrays. *Bioinformatics* **23**, 2700–2707. (doi:10.1093/bioinformatics/btm412)

7. Kauffmann, A., Gentleman, R. & Huber, W. 2009 arrayQualityMetrics--a bioconductor package for quality assessment of microarray data. *Bioinformatics* **25**, 415–416. (doi:10.1093/bioinformatics/btn647)

8. Smyth, G. K. & Altman, N. S. 2013 Separate-channel analysis of two-channel microarrays: recovering inter-spot information. *BMC Bioinformatics* **14**, 165. (doi:10.1186/1471-2105-14-165)

9. Conesa, A., Götz, S., García-Gómez, J. M., Terol, J., Talón, M. & Robles, M. 2005 Blast2GO: a universal tool for annotation, visualization and analysis in functional genomics research. *Bioinformatics* **21**, 3674–3676. (doi:10.1093/bioinformatics/bti610)

10. Bolker, B. M. 2008 *Ecological models and data in R*. Princeton, N.J: Princeton University Press.

11. Crawley, M. J. 2007 *The R book*. Chichester, England ; Hoboken, N.J: Wiley.
